# Application of a Non-Targeted Metabolomics Study in *Plasmodium berghei*-Infected Rats: Towards Unravelling Metabolic Alterations During Malaria Infection

**DOI:** 10.3390/ijms262110324

**Published:** 2025-10-23

**Authors:** Zoxolo Nokulunga Mbuli, Innocent Siyanda Ndlovu, Bubuya Masola, Samson Mukaratirwa

**Affiliations:** 1 School of Life Sciences, University of KwaZulu-Natal, Westville Campus, Durban 3630, South Africa; mbulizoxolo@gmail.com (Z.N.M.); innocent.ndlovu@mrc.ac.za (I.S.N.); 2 Non-Communicable Diseases Research Unit, South African Medical Research Council, Parow Valley 7505, South Africa; 3 One Health Center for Zoonoses and Tropical Veterinary Medicine, School of Veterinary Medicine, Ross University, Basseterre KN0101, Saint Kitts and Nevis; masolab@ukzn.ac.za

**Keywords:** *Plasmodium berghei*, malaria, metabolomics, biomarker discovery, animal model

## Abstract

Falciparum malaria is a life-threatening vector-borne disease prevalent in tropical and subtropical regions. The complexity of severe malaria demands a thorough investigation of host–parasite interactions. Twenty male Sprague Dawley rats were divided into two groups: uninfected controls and *Plasmodium berghei*-infected rats, infected via intraperitoneal injection of parasitized red blood cells. Serum samples were analysed using high-resolution untargeted Gas Chromatography–Time-of-Flight Mass Spectrometry. Metabolomic analyses revealed altered metabolites and enriched metabolic pathways. Distinct metabolite profiles were observed between infected and control groups. Infected rats showed elevated urea levels and reduced concentrations of 1,5-anhydroglucitol, D-(+)-Talose, and arachidonic acid. Pathway analysis revealed significant enrichment of the glucose-alanine cycle, alpha-linolenic acid metabolism, and linoleic acid metabolism in infected rats. Minimal enrichment was observed in arachidonic acid metabolism and lactose biosynthesis. The upregulation of the glucose-alanine cycle suggests increased gluconeogenesis in response to parasite-induced glucose depletion and energy demand. Elevated urea indicates enhanced amino acid catabolism. These findings highlight the potential of metabolomics as a diagnostic tool for malaria detection and prognosis.

## 1. Introduction

Malaria, a life-threatening vector-borne disease predominantly found in tropical and subtropical regions, continues to pose a significant global health challenge [1]. Female *Anopheles* mosquitoes are the main vectors of the disease, which claimed an estimated 569,000 lives in 2023, with the WHO African region bearing the brunt of the burden. The region accounted for 94% of global cases, translating to approximately 246 million cases [1]. Alarmingly, children under five years of age accounted for approximately 76% of all malaria deaths in this region, highlighting the vulnerability of this demographic to severe complications from the disease [2]. While uncomplicated malaria manifests with non-specific symptoms such as fever, chills, and fatigue, severe malaria presents with specific diagnostic criteria, including shock, respiratory distress, convulsions, acute kidney injury, and coma [3,4]. Among the five *Plasmodium* species that infect humans (*P. falciparum*, *P. vivax*, *P. ovale*, *P. malariae*, and *P. knowlesi*), *P. falciparum* is the most significant due to its association with severe disease and high mortality rates. The pathology of malaria is related to the parasite’s complex life cycle, initiated by the injected sporozoites in the human host that migrate to the liver and invade hepatocytes [5]. The *Plasmodium* parasite causes disruptions to host processes, specifically the erythrocytic stage where *P. falciparum* resides within the red blood cells (RBCs) and multiplies asexually during the 48 h intraerythrocytic developmental cycle (IDC) [6], using the human erythrocyte as the primary source of nutrients during development. The most common severe manifestations of malaria include cerebral malaria, acute lung injury, and acute kidney injury [7].

*Plasmodium falciparum* infection has been reported to induce systemic abnormalities involving carbohydrate metabolism [8], lipid metabolism [9], and amino acid metabolism [10]. These metabolic derangements significantly alter the host’s energy balance and nutrient utilization, complicating the clinical course of the disease. The persistent global impact of *falciparum* malaria, coupled with the emergence of multi-drug resistance [1], necessitates the development of new diagnostic techniques and therapeutic strategies. Given the complex systemic effects, a deeper understanding of the host–parasite interactions at the metabolic level is essential.

In malaria research, *P. berghei* models play a pivotal role in advancing the understanding of disease progression, efficacy of antimalarial drugs, and testing of vaccine candidates [11,12]. This rodent parasite is widely used in animal models due to its ability to replicate several key features of human malaria, including anemia, immune response, splenomegaly, and cerebral-like symptoms [13,14]. These characteristics make *P. berghei* an invaluable tool for preclinical studies aimed at elucidating the host interaction and identifying novel therapeutic strategies [11,15]. Advances in malaria research using high-resolution metabolomic methods have enabled the enhanced understanding of host–parasite interactions, with the aim of identifying novel treatments and diagnostic strategies [16].

There is a paucity of information on the extent to which metabolic activity of *Plasmodium* spp. impacts the host metabolism [17]. Additionally, an in-depth understanding of how the parasite and the host metabolism are interconnected will aid in determining the optimal metabolic steps to be taken for diagnosis and drug discovery. According to Yu et al. (2021) [18], metabolomics analyses from in vitro studies have attempted to build the foundation for stage-specific metabolic profiles in *Plasmodium* infection, which results in a comprehensive understanding of *Plasmodium* biology at the cellular level in the definitive host.

The aim of this study is to apply a non-targeted metabolomics approach and profile metabolite changes in Sprague Dawley rats experimentally infected with *P. berghei* and profile metabolic alterations and pathways that are mostly impacted and/or enriched during the infection and identify potential biomarkers for diagnosis of infection or prognosis of disease.

## 2. Results

### 2.1. Parasitaemia

**Figure 1:**
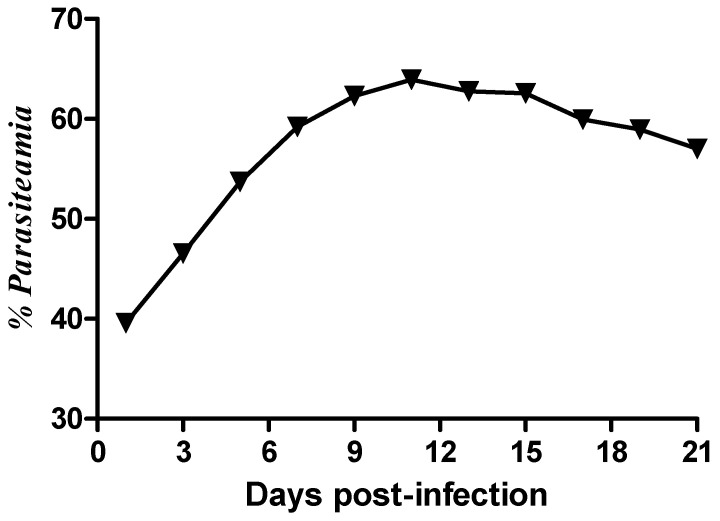
Percent parasitemia of *Plasmodium berghei* in experimentally infected Sprague Dawley (SD) rats for a 21-day period when animals were sacrificed post-infection.

The proportion of parasitized RBCs (% parasitemia) of the experimental animals was determined from blood smears taken every two days until day 21 pi. The percentage parasitemia showed a steady rise from day 3 pi and reached a peak between day 11 and 12 pi (Figure 1).

### 2.2. Metabolite Changes Between Days of Sacrifices Post Infection

**Figure 2:**
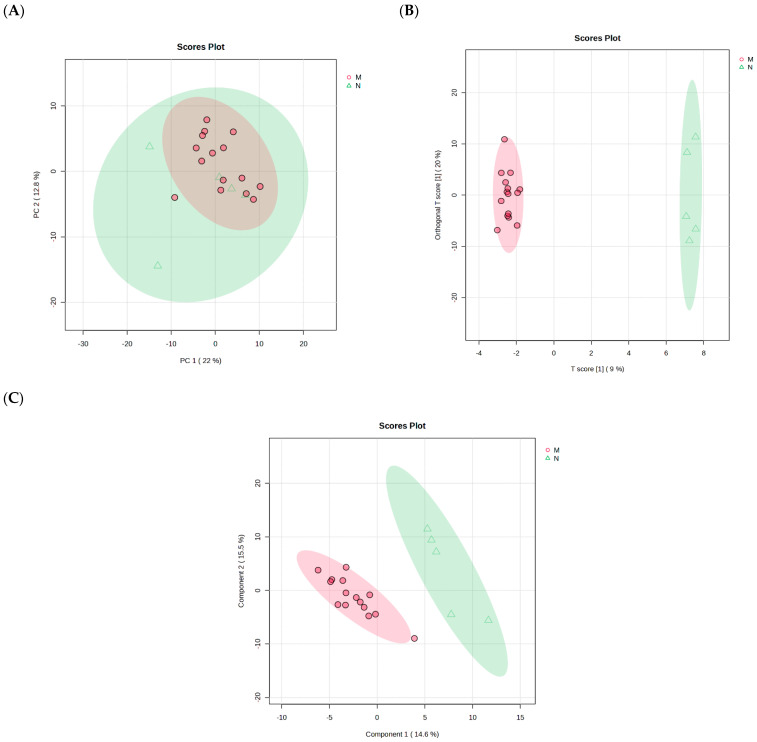
Illustration of the natural discrimination of metabolic profiles found in the two test groups. PCA (**A**), PLS−DA (R^2^ = 0.907, A = 0.90, Q^2^ = 0.41) (**B**) and OPLS−DA (R^2^X = 0.08, R^2^Y = 0.72, Q^2^ = 0.46) (**C**) scatter plots differentiating the potential metabolites present between the *Plasmodium berghei*-infected group and uninfected control group. (Triangles within the green oval represent the control group, while circles within the red oval represent the *Plasmodium berghei*-infected group).

**Figure A1:**
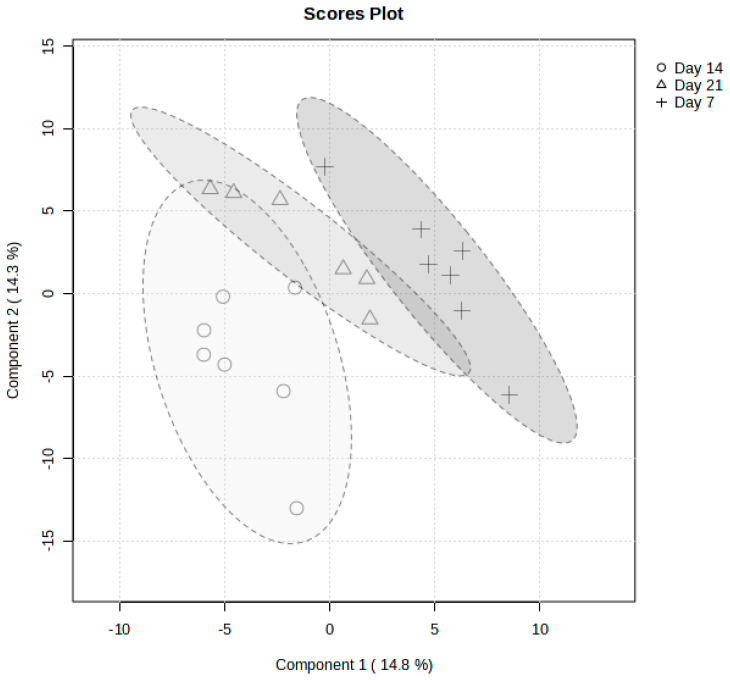
Discrimination of metabolic profiles across sacrifice days during *P. berghei* infection using PLS−DA.

From the analyzed serum samples, a total of 744 metabolites were identified (Figure 2). The PCA showed no clear separation of metabolic changes among experimental rats sacrificed at day 7, 14, and 21 post-infections (Appendix A Figure A1). Additionally, the PLS-DA depicted moderate separation among the days of sacrifice pi with component 1 and component 2 explaining 12.8% and 14.3%, respectively (Appendix A Figure A1). According to the ANOVA multiple comparison analysis, D-glucose and DL-arabinose were the only identified metabolites that were statistically significant (*p* ≤ 0.05) among the 744 identified (Appendix A Table A1).

**Figure A2:**
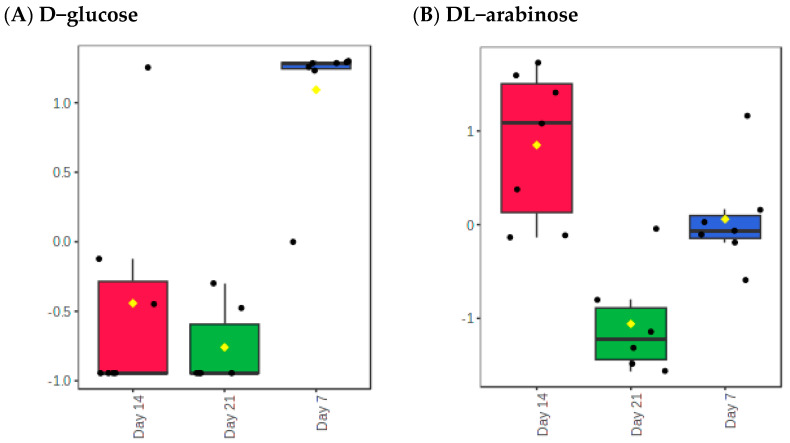
Boxplot showing metabolite concentration change of D−glucose and DL−arabinose in different days of sacrifice during *Plasmodium berghei* infection.

A temporal decline in D-glucose concentration was observed from day 7 to day 21 post-infection (Appendix A Figure A2A). Statistically significant differences in D-glucose levels were detected between day 7 and day 14, as well as between day 7 and day 21 post-infection (Appendix A Table A1). In contrast, DL-arabinose levels increased from day 7 to day 14, followed by a decrease on day 21 post-infection (Appendix A Figure A2B). Significant differences in DL-arabinose concentration were observed across all pairwise comparisons: day 7 vs. day 14, day 14 vs. day 21, and day 7 vs. day 21 (Appendix A Table A1). Due to the absence of statistically significant differences across the time points (Days 7, 14, and 21 post-infection), the data were subsequently pooled into two groups, *P. berghei*-infected and non-infected control, to assess overall metabolite alteration associated with infection status.

### 2.3. Pooled of Plasmodium berghei-Infected and Non-Infected Control Group

From the PCA, there was no clear separation between the *P. berghei*-infected and the non-infected control group and the PCs where PC1 explained 22% variation and PC2 explained 12.8% of total variance (Figure 2A). To further determine the separation between the two groups, a PLS-DA plot was used, and it depicted a clear separation between the groups where Component 1 and 2 at 45% and 15.5% of total variance, respectively (Figure 2B). The OPLS-DA plot further showed separation with PC1 accounting for 9% and PC2 accounting 20% of the total, respectively (Figure 2C). The different distribution patterns of potential metabolites between the *P. berghei*-infected and non-infected control group observed indicate the applicability of the two models and changes in serum metabolites composition in *P. berghei* rats. Additionally, from the PLS-DA, the goodness-of-fit parameters (R2Y; R2X and Q2Y) depicted that the model was not overfitting and reliable.

### 2.4. Identification of Differential Metabolites

**Figure 3:**
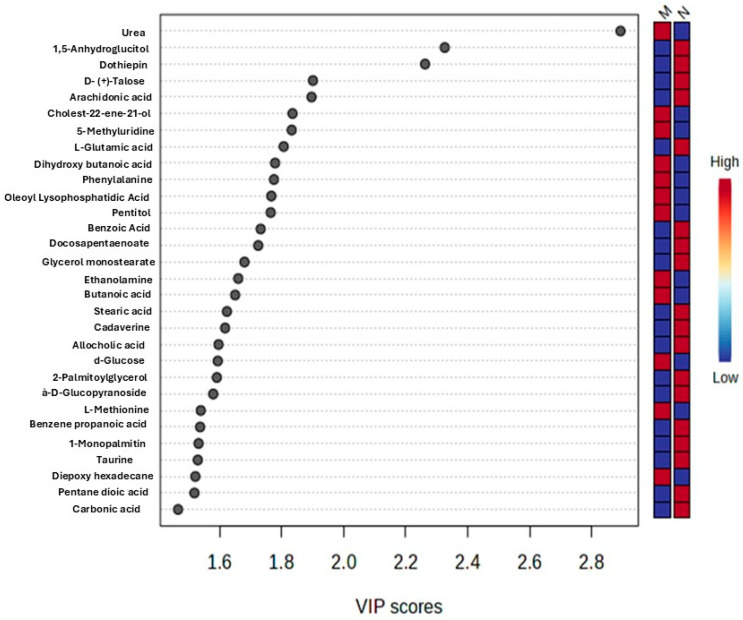
Illustration of the top 30 important metabolites ranked by the PLS-DA VIP score. The mini heatmap on the right indicates their concentration variations within the different groups. Where M = *Plasmodium berghei*-infected and N = non-infected control group of Sprague Dawley rats.

**Figure 4:**
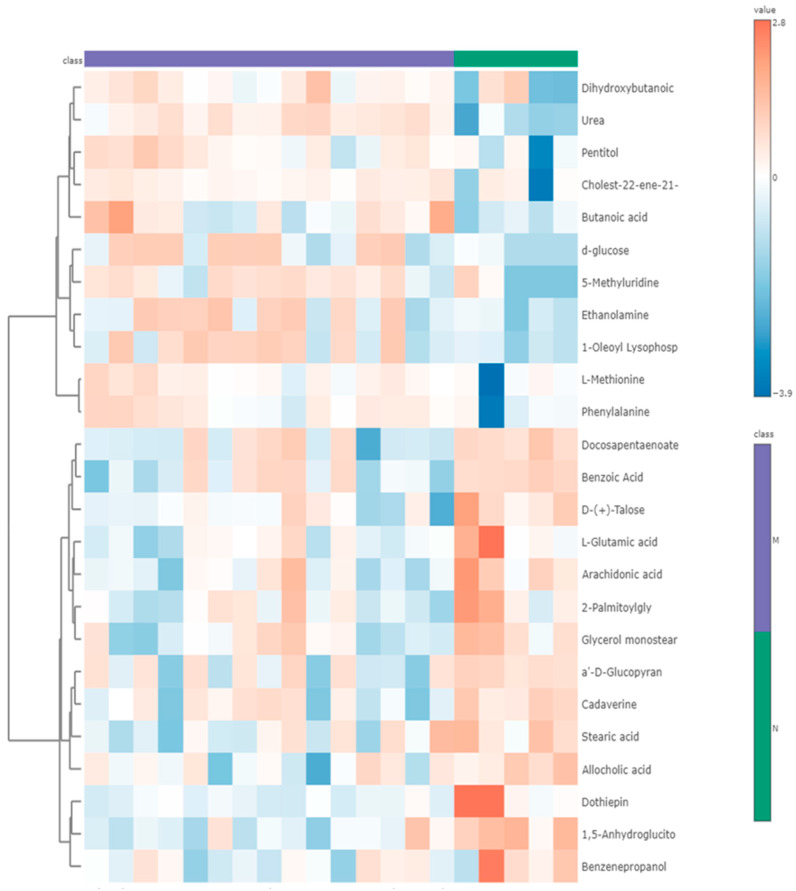
Heatmap analysis showing hierarchical clustering of metabolic data from the *Plasmodium berghei*-infected and non-infected control group based on the top 30 ranked potential biomarkers. Value-normalized intensity of each metabolite, Purple = Infected group; Green = non-infected control group. Orange-dark orange represents metabolites with increased intensity and light-dark blue indicates metabolites with reduced intensity.

Significant metabolites were identified at an absolute cut-off value of the coefficient and Variable Importance in Projection (VIP) ≥ 1 when performing PLS-DA. These were then considered as the most significant metabolite and potential biomarkers that differentiate the control and malaria-infected group. The top 30 metabolites were the most significant biomarkers (Figure 3). The VIP plot depicts the differing concentrations of all identified potential biomarkers between *P. berghei*-infected rats and non-infected rats. The metabolic classes of the identified potential biomarkers included organic compounds, carbohydrates, xenobiotics, fatty acids, pyrimidine nucleosides, amino acids and carboxylic acid (Table 1). Urea was upregulated in the *P. berghei*-infected group compared to the non-infected control group, with a VIP score of 2.892. Moreover, the metabolites; 1,5-anhydroglucitol, dothiepin, D-(+)-Talose, and arachidonic acid were downregulated in the *P. berghei*-infected group when compared to the non-infected control group. Two predominant hierarchal clusters were observed on a heatmap depicting the most intense changes in metabolites between the infected and non-infected control group (Figure 4). Additionally, the top cluster contains metabolites such as urea, pentitol, which are of higher relative abundance in the infected group and lower in the control. Cluster two included metabolites like l-glutamic acid, dothiepin and arachidonic acid. And these were more abundant in control. Cluster one was dominated by amino acids derivatives and small organic acids, potentially reflecting changes in energy metabolism and amino acids catabolism.

### 2.5. Metabolic Pathway and Enrichment Analysis

**Figure 5:**
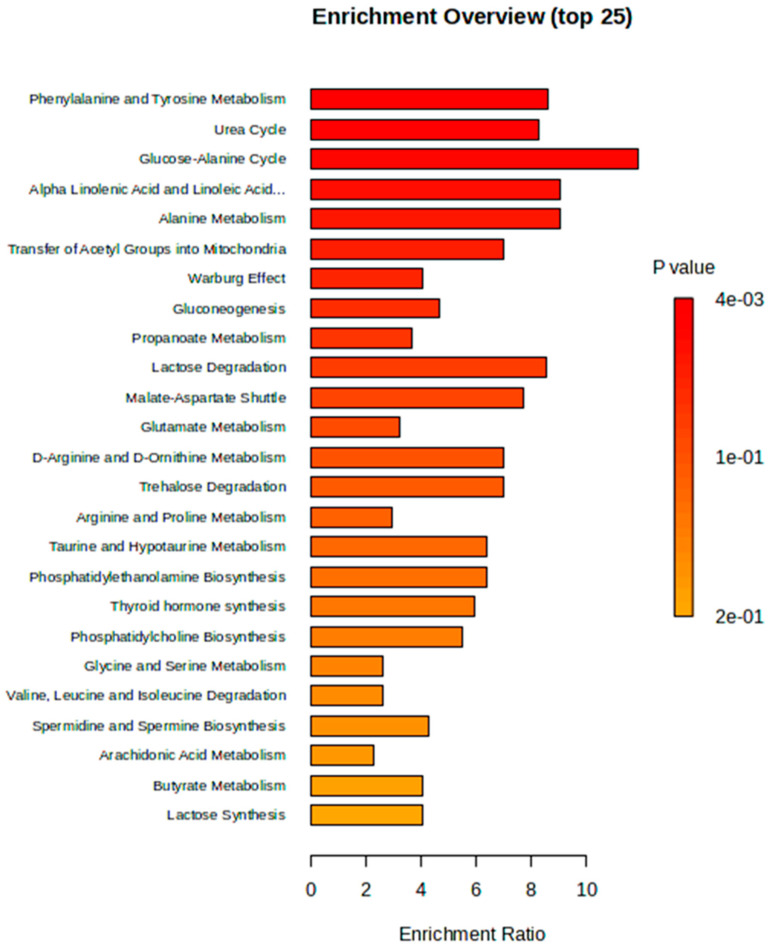
Illustration of metabolic pathways connected to the top 30 identified potential metabolites in the study groups. The horizontal bars represent the metabolic pathways most impacted during *Plasmodium berghei* infection in Sprague Dawley male rats. Alpha Linolenic Acid and Linoleic Acid…. = Alpha Linolenic Acid and Linoleic Metabolism.

**Figure 6:**
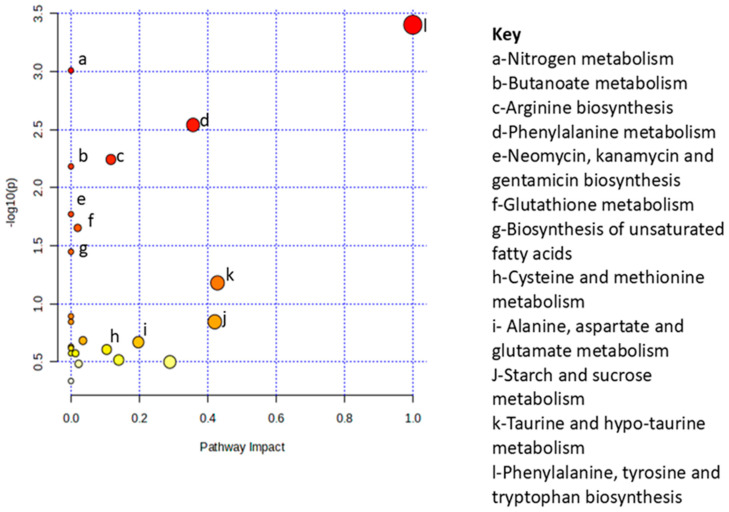
MetPA indicating matched pathways. Pathway enrichment versus log10 with *p* < 0.05 cut-off comparison of *Plasmodium berghei*-infected and non-infected control Sprague Dawley male rats. All matched pathways represented as circles, where the size of the node correlates with the enrichment ratio. Red and yellow depict *p*-values from highest to lowest. The size and color of each node are based on the impact value and *p*-value, respectively. The most impacted pathways, having high statistical significance scores, are labelled with alphabets, with the key descriptions on the right side of the graph.

Metabolite set enrichment analysis (MSEA) was used to identify and interpret significantly enriched and biologically relevant patterns that were in the quantitative metabolic data between the two study groups (Figure 5). From the top 30 differentiating metabolites (Appendix A Table A2), glucose-alanine cycle, alpha linolenic acid and linoleic acid metabolism, alanine metabolism, phenylalanine and tyrosine metabolism, and the urea cycle were pathways that were significantly enriched during *P. berghei* infection (Figure 5). While some of the least enriched pathways during malaria infection include the arachidonic acid metabolism, butyrate metabolism and lactose synthesis. To determine metabolic pathways that were impacted during infection, Metabolic Pathway Analysis (MetPA) was used. Based on the MetPA, metabolome overview was produced, showing phenylalanine, tyrosine and tryptophan biosynthesis as the most significant and highly impacted metabolic pathway during malaria infection. Additionally, nitrogen metabolism, phenylalanine metabolism and arginine biosynthesis were less impacted but significant metabolic pathways in *P. berghei* infected rats (Figure 6).

### 2.6. Determination of Potential Biomarkers

**Figure 7:**
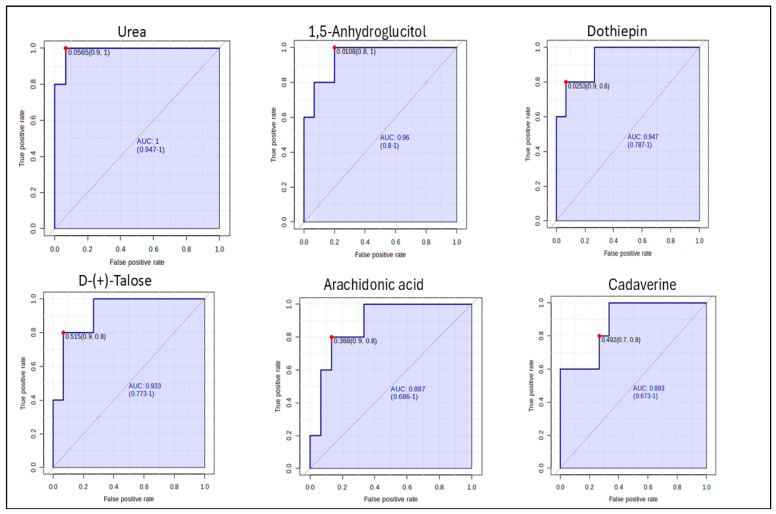
The Receiver Operating Characteristic (ROC) analysis of potential biomarkers for differentiating the *Plasmodium berghei*-infected group from the control. (AUC—Area Under the Curve).

A Receiver Operating Characteristic (ROC) analysis was performed to determine the predictive value of the identified metabolites (Figure 7). The criteria were set at 0.9 ≤ AUC ≥ 1 being considered excellent. Urea, 1,5-Anhydroglucitol, dothiepin and D-(+)-Talose showed excellent diagnostic potential for *P. berghei* infection, with an AUC of 1; 0.96; 0.947 and 0.933 respectively. Furthermore, the top 6 potential biomarkers all portrayed a good predictive value of more than 0.8. However, the ROC analysis showed Urea to have the highest diagnostic ability with an AUC value of 1 (*p* < 0.05) and confidence interval of 94.7–100.

## 3. Discussion

*Plasmodium berghei* parasitemia observed in our study showed a steady rise, with a notable peak observed between days 11 and 12 pi. This consistent increase in parasitemia agrees with previous studies by Umo et al. (2017) [19] and Paul et al. (2015) [20], depicting a progressive parasite growth and replication of the parasite over the course of the experiment. A study by Sibiya et al. (2016) [21] reported a peak in parasitemia around day 14 pi, indicating a commonality in the development of infection dynamics across experimental models. According to Krugliak et al. (2002) [22] once the parasite has invaded the red blood cells, the parasite triggers significant alterations to the host cell, facilitating the regulated exchange of metabolites. Moreover, the parasite induces new permeability pathways on the host cell membrane to optimize the influx and efflux of specific compounds [20,23]. Additionally, the parasite initiates a catabolic process in which hemoglobin from the erythrocyte cytoplasm is ingested and broken down into its amino acid components within an acidic vacuole [24].

Our study employed a non-targeted metabolomics approach to elucidate the metabolic alterations occurring in male SD rats infected with *P. berghei*. The rationale for using rodent models in the study of human diseases stems from the genetic and physiological similarities shared between these species during *Plasmodium* spp. infection [25]. Through the application of metabolomics, we identified several metabolic changes associated with *P. berghei* infection, encompassing a diverse array of organic compounds, including carbohydrates, xenobiotics, fatty acids, pyrimidine nucleosides, amino acids, and carboxylic acids. This breadth of identified metabolites underscores the complex nature of the metabolic dysregulation caused by *P. berghei* infection. The metabolite alterations observed in our study closely resemble those reported in *Plasmodium falciparum*-infected humans, particularly with respect to disruption in energy metabolism [26] and lipid profile modification associated with parasite development [27]. Similar metabolic perturbations during malaria infection have been documented by Na et al. (2020) [28], who identified distinct metabolic phenotypes differentiating non-infected controls from individuals infected with *P. vivax* and *P. falciparum*.

Metabolomics studies have significantly advanced our understanding of the metabolic alterations induced by *P. falciparum* infection [17]. In the present study, the univariate and multivariate statistical techniques distinguishing the *P. berghei*-infected and non-infected control groups based on complex metabolomic profiles and significant metabolite separation observed suggest that the *P. berghei* infection causes metabolite shift in infected hosts. Urea concentration was significantly elevated in *P. berghei*-infected compared to the uninfected control rats, supporting findings by [27].

In humans as well as in rodents, urea, a nitrogenous waste product generated during protein metabolism in the liver, is filtered from the bloodstream by the kidneys and excreted in urine [29]. Elevated urea levels in serum serve as a marker for acute kidney injury (AKI), with studies indicating that AKI occurs in up to 45% of individuals with severe malaria, associated with mortality rates as high as 74% when dialysis is required but unavailable [10,30]. The pathological impact of malaria in children and pregnant women includes renal and hepatic dysfunction. Moreover, renal impairment is marked by elevated levels of creatinine, urea, and certain serum electrolytes, whereas hepatic dysfunction is indicated by increased liver enzyme activity [31].

Our results are in accordance with the study conducted by Akanbi (2015) [31], who reported an increase in the urea concentration in children with malaria when compared to the control group. According to Zaki et al. (2013) [32], the elevation of urea concentration during malaria infection could be due to parasite sequestration to the renal microvascular bed. Therefore, we can speculate that indeed malaria has profound effects on the renal functions of their host during infection, as observed by Akanbi (2015) [31]. These findings therefore underscore the significance of monitoring renal function in infected patients to immediately manage the risks of potential kidney failure or complications. Another factor in the elevation of urea levels may be the increased activity of the glucose–alanine cycle and related gluconeogenesis due to an increased energy demand brought about by infection. Malaria infection results in host hypoglycemia which, according to Ramos et al. (2022) [33], may be due to inhibition of hepatic gluconeogenesis by labile heme from RBC lysis, glucose consumption by the parasite, and illness-induced anorexia. Thus, glucose levels need to be monitored in addition to increased levels of urea, which result from disposal of amino acid nitrogen prior to gluconeogenesis. It is no coincidence that severe malaria results in cerebral disturbances, considering dependence of the brain on glucose as a respiratory fuel. Furthermore, our study suggests an increase in levels of phenylalanine, which may contribute to cerebral disturbances in severe malaria, since elevated levels of phenylalanine are, in themselves, toxic to brain cells [34] and will also cause disturbances in the balance of brain neurotransmitters such as dopamine, noradrenaline and serotonin.

Other metabolites that were significantly impacted or changed during infection in our study included carbohydrates such as 1,5-Anhydroglucitol, d-talose, and pentitol. According to Planche et al. (2005) [35], carbohydrate disorders such as acidosis and hypoglycemia are amongst the key indicators of disease severity in individuals infected with *P. falciparum.* These identified carbohydrate metabolites were downregulated during *P. falciparum* infection. According to Selvin et al. (2016) [36], in a normal state, the monosaccharide 1,5-Anhydroglucitol levels in the blood are typically high. Moreover, 1,5-Anhydroglucitol is filtered by the glomeruli and reabsorbed in the renal tubule and small amounts are excreted in the urine. In our study, 1,5-Anhydroglucitol was downregulated during infection when compared to the control group. It can be speculated that the excretion of this metabolite was high during *P. falciparum* infection. While this metabolite has been proposed in diabetes studies to be a potential biomarker to explore glycemic control [37], it is imperative to further explore this in cases of malaria and diabetes comorbidity.

The identified metabolites in our study were both directly and indirectly associated with multiple metabolic pathways, and these included urea cycle, amino acid metabolism, glutathione metabolism, glucose alanine cycle, TCA cycle, and arginine biosynthesis. Amongst the groups of metabolites, amino acids and lipids have been reported to be changed by *P. falciparum* infection [35]. The metabolic pathway detected and highly impacted in our study was phenylalanine, tyrosine, and tryptophan biosynthesis. Additionally, the amino acid metabolites that were associated with this pathway included L-phenylalanine and tyrosine. L-glutamate was detected in multiple metabolic pathways in the current study and was downregulated during infection when compared to the control group. The pathophysiology of severe *falciparum* malaria is closely associated with disruptions in amino acid metabolism. Notable biochemical abnormalities observed in *falciparum* malaria include L-arginine deficiency, which correlates with endothelial dysfunction [38], elevated levels of L-lactate and alanine linked to metabolic acidosis [39,40], increased phenylalanine [38,41], and heightened levels of tryptophan metabolites [42], all of which are associated with disease severity. Furthermore, low glutamine levels may contribute to the modulation of oxidative stress [43], therefore impacting immune cells since they use glutamine as source of energy.

The pathways related to glutathione metabolism are significant since glutathione plays a crucial role in detoxification processes within the body. Its dysregulation could indicate a compromised state of the host’s antioxidant defences, leaving it more susceptible to oxidative stress caused by the parasite.

Metabolic pathways that were less impacted but significant during infection in our study included biosynthesis of unsaturated fatty acids. This biosynthesis plays an important role in parasite infection. Moreover, these metabolites are essential components of the parasite’s cell membrane, playing a crucial role in maintaining their fluidity, stability, and interactions with the host environment [44]. According to [45], fatty acids are intrinsic components for adaptation of the parasite to different environments during their lifecycle and in serving as the energy source. The perturbation of the identified fatty acids we observed in our study may be attributed to the host’s attempt to meet increased energy demands or to produce components necessary for membrane synthesis in response to infection.

In this study, adding to the observed changes in lipids, particularly the perturbation in arachidonic acid, the observed changes in this metabolite can reflect underlying oxidative and enzymatic perturbations linked to malaria infection. Arachidonic acid, an omega-6 polyunsaturated fatty acid found in cell membrane [46], is highly susceptible to lipid peroxidation under oxidative stress, which then results in the formation of reactive aldehydes such as 4-hydoxynonenal (4-HNE). This aldehyde is a known mediator of cellular damage, inflammation, and signaling molecules [46,47]. Previous studies have reported that hemozoin pigment induces peroxidation of arachidonic acid, generating 4-HNE, which will then lead to the disruption of monocyte motility, immune cell function, and cytoskeleton integrity [47]. In our study, we speculate that this can be the plausible mechanism for the observed changes in the concentration of arachidonic acid. A study conducted by Skorokhod and Berrera (2021) [46], reported that 4-HNE binds to CYP4F11 in monocyte, thereafter, reducing its ω-hydroxylation activity toward arachidonic acid, which leads to the elevation of inflammatory eicosanoids and altered immune response. Therefore, the changes in these metabolic pathways provide mechanistic insights into how changes in lipids metabolism and arachidonic acid dysregulation may contribute to the pathogenesis in malaria.

A limitation of this study was the relatively small number of experimental animals, which, although sufficient to demonstrate clear metabolic differences, may reduce the statistical power. Therefore, future studies with larger samples size will be important to strengthen and validate these findings

## 4. Materials and Methods

### 4.1. Ethical Consideration

The study was conducted at the University of KwaZulu-Natal Biomedical Resource Unit located at the Durban Westville Campus, South Africa. All procedures and protocols were reviewed and approved by the University of KwaZulu-Natal Animal Research Ethics Committee, under the protocol reference number: AREC/008/019D.

### 4.2. Experimental Design

**Figure 8:**
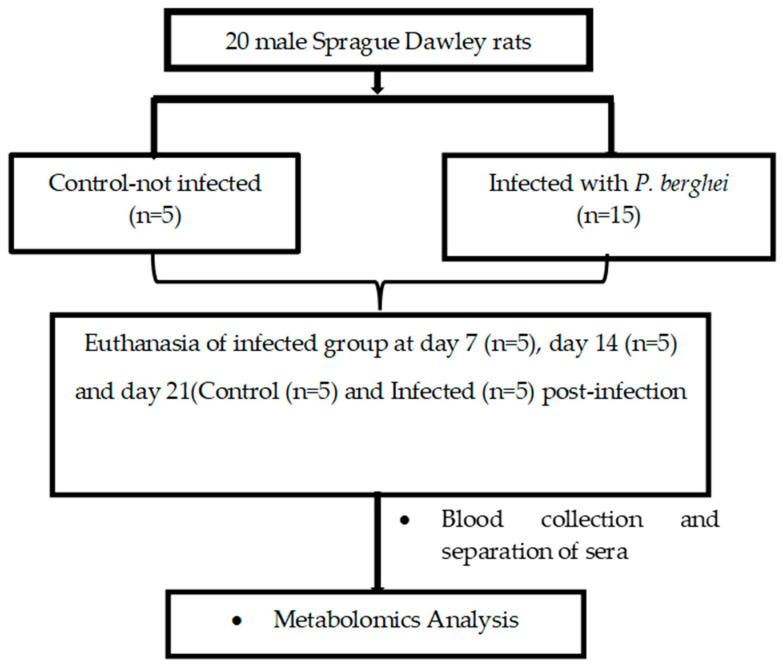
Flow diagram of experimental design.

Twenty Sprague Dawley rats (80–120 g) were divided into two groups; Group 1 (Control-non-infected; n = 5) and Group 2 (Experimental-*P. berghei* infected; n = 15) (Figure 8). Percentage parasitaemia and body weight were measured and recorded every second day post-infection in the experimental group until day 21 post-infection and five rats from the *P. berghei*-infected and the control group were euthanized on day 21 post infection.

### 4.3. Infection of SD-Rats with Plasmodium berghei Parasite

Chloroquine-sensitive *Plasmodium berghei*, obtained from the University of Cape Town’s Clinical Pharmacology Division, South Africa, was used in this study. A small group (n = 3) of stock male Sprague-Dawley rats (90–150 g) were initially infected via a single intraperitoneal injection using a 25G needle containing 10^5^ *P. berghei*-parasitized red blood cells suspended in freshly prepared phosphate-buffered saline (PBS, pH 7.4), as described by Diehl, et al. (2001) [48]. Once a stable infection was established in these stock rats, they were euthanized via ISOFOR inhalation, and blood was collected through cardiac puncture into serum and serum-gel separator tubes. The blood was centrifuged for 5 min using a Sorvall Legend Micro 17 Microcentrifuge (ThermoFisher Scientific, Waltham, Massachusetts, USA), and the separated serum was stored at −80 °C for future use. The collected infected blood was subsequently used to induce infection in experimental male Sprague-Dawley rats via a single intraperitoneal injection of 10^5^ parasitized red blood cells, following the method described by Beaudoin, (1977) [49]. Control animals received an injection of PBS vehicle alone. Infection was confirmed through microscopic examination of thin tail blood smears stained with Giemsa.

### 4.4. Assessment of Parasitemia

Parasite density in experimental animals was measured every two days to minimize animal distress. Blood was collected from the caudal vein of each rat and the preparation of thin blood smears and staining procedures were followed according to Eberhard and Lammie (1991) [50] The thin smear slides were fixed with methanol (Merck chemicals (PTY) Ltd., Modderfontein, South Africa) and stained with 20% Giemsa (Merck chemicals (PTY) Ltd.). After staining, the slides were air dried and observed using a Zeiss Primo Star Binocular Microscopeat ×100 oil immersion objective. Five microscope fields were counted, and calculations were done, using the equation below to determine percentage parasitaemia.

(1) $$\%\;Parasitaemia=\frac{Number\;of\;parasitized\;RBC\;counted}{Number\;of\;RBC\;counted\;on\;five\;microscope\;fields}\times 100$$

For the experimental rats, percentage parasitaemia greater than or equal to 20% for each rat was considered as stable *P. berghei* infection and qualified the rat to be included in the experimental group.

### 4.5. Terminal Studies

At day 7 (n = 5), 14 (n = 5) and 21 (n = 5) post-infection and day 21 (n = 5) experimental rats (Figure 1) were euthanized using ISOFOR inhalation for 3 min in an anaesthetic chamber. Blood was collected via cardiac puncture into 10 mL tubes containing clotting activator (dya cgel and clot activator, Terumo^®^ Venosafe^®^, Terumo, Tokyo, Japan). The blood was then centrifuged in a microprocessor controlledHeraeus Labofuge 200 centrifuge (Thermo-scientific, Wiltham, MA, USA) at 132 rpm for 15 min at 4 °C and thereafter, the sera were collected and stored at −80 °C in the Bio Freezer (Snijders Scientific, Tilburg, The Netherlands) until they were transported to the North-West University Centre for Human Metabolomics (Potchefstroom, South Africa) for analysis using Untargeted GC/TOF-MS Metabolomics.

### 4.6. Serum Analysis

Twenty serum samples (n = 5-Control group; n = 15-Experimental group) were transferred to the North-West University Centre for Human Metabolomics (Potchefstroom, South Africa). The serum samples were stored in 2 mL Eppendorf tubes (Merck, Johannesburg, South Africa) and placed in an upright position into an ice-filled ice box. The uninfected Control group sera were labelled as N1-5 while the *P. berghei* experimental group were labelled as M1-15. The whole metabolome analysis was applied to the samples. As internal standard, 50 uL of 3-phenylbutyric acid (100 ppm) was added to 50 uL of each sample. Proteins were precipitated by adding 300 uL of ice-cold acetonitrile followed by incubating the samples on ice for 10 min. Sample vials were then centrifuged at 10,000 rpm for 10 min (4 °C), and the supernatant was collected and dried under nitrogen. The dry extract was then derivatized with 25 uL of methoxyamine hydrochloride in pyridine (20 mg/mL) at 50 °C for 60 min, and trimethylated with 40 uL of BSTFA containing 1% TMCS at 60 °C for 60 min. Samples were extracted and derivatized in a random order and sample preparation was followed as escribed by Swanepoel et al. (2020) [51].

### 4.7. Bench Top Gas Chromatography Coupled to a Time-of-Flight Mass Spectrometer (BT GC-TOFMS) Untargeted-Approach

**BT GC-TOFMS analysis.** A Pegasus BT GC-TOFMS (Bench Top Gas Chromatography coupled to a Time-Of-Flight Mass Spectrometer) (Leco Corporation, St. Joseph, MI, USA) equipped with an Agilent 7693A automatic liquid sampler (Leco Corporation, St. Joseph, MI, USA) was used for chromatographic analysis of the derivatized samples. One microlitre of serum sample was randomly injected at a slit ratio of 3 with helium at a constant flow as the carrier gas for 1 mL/min for the entire run. The transfer line temperature was at 270 °C, for the entire run. The GC oven temperature was initially programmed at 70 °C for 1 min where after it was increased at 10 °C/min to 320 °C where it was kept for 3 min.

**Peak identification.** Leco Corporation ChromaTOF software (version 4.72) was used for peak finding and mass spectral deconvolution at an S/N ratio of 300, with a minimum of two apexing peaks. Using the mass fragmentation patterns generated by the MS, together with their respective GC retention times, the identities of these peaks were determined by comparing them to commercially available NIST spectral libraries (mainlib, replib), with a similarity of 700 (70%) required for a name to be assigned to a peak Schymanski et al. (2014) [52].

**Raw data processing.** Raw data, including peak areas and annotations for each sample, were exported to Microsoft Excel. Contaminant compounds were identified by comparing the compounds detected in the extraction blank to those detected in the samples. Peaks detected in the extraction blank were deleted from the dataset if its mean concentration in the samples were 3 times or less than that in the blanks [53]. Samples were quantified using the detected area of the internal standard to get to a relative concentration in ng/ul sample.

### 4.8. Statistical Analysis

Data were analysed using an online software, MetaboAnalyst version 6.0. Metabolite intensity was presented as median with Inter Quartile Range (IQR). Data were normalised via log transformation and auto-scaling. An unsupervised Partial Component Analysis (PCA) was used to distinguish between a pattern or separation among the metabolites. This was followed up with a supervised method, Partial Least Squares-Discriminant Analysis (PLS-DA), that used multivariate regression techniques to determine whether there is a clear separation within the sample metabolites. To determine other biological variations and to improve the separation of the sample metabolites, a supervised Orthogonal Partial Least Squares Discriminant Analysis (OPLS-DA) method was performed. The goodness-of-fit parameters (R2Y; R2X and Q2Y) were calculated. To further evaluate the identified potential metabolic biomarkers discovered in the experimental samples, a receiver-operating curve analysis was conducted. In addition, the AUC (Area under the curve) was utilised to determine the diagnostic accuracy of the biomarkers where 0.8 < AUC > 0.9 was considered good and 0.9 ≤ AUC ≥ 1 was considered excellent using MetaboAnalyst. One way analysis of variance was performed to determine statistical significance of the difference in the levels of potential biomarkers, where *p* ≤ 0.5 were considered significant. To determine the Metabolite Set-Enrichment Analysis (MSEA) and MetPath modules on MetaboAnalyst were performed.

## 5. Conclusions

The metabolic profiling of *P. berghei*-infected male SD rats revealed significant alterations compared to non-infected controls, characterized by up- and downregulated metabolites across various metabolic pathways, which is comparable to *P. falciparum* infection in humans. These changes highlight the systemic impact of *P. berghei* parasite on the host, reflecting increased protein catabolism, altered glucose metabolism, dysregulation of inflammatory responses, and shifts in cellular growth and stress pathways. Enrichment of glucose-alanine cycle indicates a shift toward gluconeogenesis as the host attempts to keep glucose homeostasis under the metabolic stress of infection. Moreover, this enrichment means an increased energy demand and nitrogen waste handling during malaria infection. The findings of our study demonstrate that *P. berghei* is a valuable model organism for investigating therapeutic interventions against malaria, particularly through metabolomics-based approaches. Moreover, metabolic profiling has the potential to serve as a sensitive diagnostic approach for detection and prognosis of *Plasmodium* infection.

## Figures and Tables

**Table 1:** The top 30 identified metabolites based on the PLS-DA VIP, *p*-value, FDR score causing the differentiation of the *Plasmodium berghei*−infected from the non-infected control group and with KEGG, HMDB and PubChem numbers.

Query	Class	VIP	*p*−Value	FDR	HMDB	PubChem	KEGG
Urea	Organic compound	2.8929	7.52 × 10^−7^	0.000157	HMDB0000294	1176	C00086
1,5-Anhydroglucitol	Carbohydrate	2.3261	0.000634	0.066291	HMDB0002712	64960	C07326
Dothiepin	Xenobiotic	2.2627	0.001014	0.070658	None	None	None
D−(+)−Talose	Carbohydrate	1.9011	0.008721	0.28696	None	None	None
Arachidonic acid	Fatty acid	1.8966	0.008919	0.28696	HMDB0001043	444899	C00219
Cholest-22-ene-21-ol	Xenobiotic	1.8353	0.011972	0.28696	None	None	None
5-Methyluridine	pyrimidine nucleoside	1.8326	0.012128	0.28696	HMDB0000884	445408	None
L−Glutamic acid	Amino acid	1.8068	0.013658	0.28696	HMDB0000148	33032	C00025
Dihydroxy butanoic acid	Fatty acid	1.7791	0.015475	0.28696	None	None	None
Phenylalanine	Amino acid	1.7754	0.015729	0.28696	HMDB0000159	6140	C00079
Oleoyl Lysophosphatidic Acid	Fatty acid	1.7668	0.016337	0.28696	None	None	None
Pentitol	Carbohydrates	1.7649	0.016476	0.28696	HMDB0000508	None	C00474
Benzoic Acid	Organic compound	1.7326	0.018952	0.29268	HMDB0001870	243	C00180
Docosapentaenoate	Fatty acid	1.7246	0.019605	0.29268	HMDB0001976	6441454	None
Glycerol monostearate	Fatty acid	1.6804	0.023575	0.31479	None	None	None
Ethanolamine	Organic compound	1.6603	0.025571	0.31479	HMDB0000149	700	C00189
Butanoic acid	Fatty acid	1.6503	0.026609	0.31479	HMDB0000039	264	C00246
Stearic acid	Fatty acids	1.6238	0.029544	0.31479	HMDB0000827	5281	C01530
Cadaverine	Organic compound	1.6179	0.030226	0.31479	HMDB0002322	273	C01672
Allocholic acid	Organic compound	1.5967	0.032792	0.31479	HMDB0000505	160636	C17737
d−Glucose	Carbohydrate	1.5942	0.033103	0.31479	HMDB0000122	5793	C00031
2-Palmitoylglycerol	Fatty acid	1.5913	0.033473	0.31479	None	None	None
à−D−Glucopyranoside	Carbohydrate	1.5795	0.034993	0.31479	None	None	None
L-Methionine	Amino acid	1.5393	0.040596	0.31479	HMDB0000696	6137	C00073
Benzene propanoic acid	Carboxylic acid	1.5373	0.040896	0.31479	HMDB0000158	6057	C00082
1−Monopalmitin	Fatty acid	1.5324	0.041622	0.31479	None	None	None
Taurine	Amino acid	1.5298	0.042014	0.31479	HMDB0000251	1123	C00245
Diepoxy hexadecane	Xenobiotic	1.5219	0.043225	0.31479	None	None	None
Pentane dioic acid	Organic compound	1.519	0.04368	0.31479	HMDB0000661	743	C00489
Carbonic acid	Xenobiotic	1.4662	0.04578	0.31479	HMDB0000596	C00288	None

HMDB = Human Metabolome Database, KEGG = Kyoto Encyclopedia of Genes and Genome.

## Data Availability

The original contributions presented in the study are included in the article; further inquiries can be directed to the corresponding author.

## Abbreviations

The following abbreviations are used in this manuscript:

*P*	Plasmodium
SD	Sprague Dawley
p.i.	post-infection
RBCs	red blood cells
IDC	intraerythrocytic developmental cycle
IQR	Inter Quartile Range
BT GC-TOFMS	Bench Top Gas Chromatography coupled to a Time-Of-Flight Mass Spectrometer
PCA	Partial Component Analysis
PLS-DA	Partial Least Squares-Discriminant Analysis
OPLS-DA	Orthogonal Partial Least Squares Discriminant Analysis
AUC	Area under the curve
MSEA	Metabolite Set-Enrichment Analysis
VIP	Variable Importance in Projection
MetPA	Metabolic Pathway Analysis

## Appendix A

**Table A1:** ANOVA analysis of metabolite changes between the three days of sacrifice post *Plasmodium berghei* infection in the SD-rats.

Metabolite	f-Value	*p*-Value	FDR	Fisher’s LSD
D-glucose	19.263	4 × 10^−5^	0.008927	Day 7–Day 14; Day 7–Day 21
DL-Arabinose	13.941	0.000253	0.027114	Day 14–Day 21; Day 14–Day 7; Day 7–Day 21

**Table A2:** Metabolic pathways linked to the top 30 significant metabolites between the control and the *Plasmodium berghei* infected group.

Metabolic Pathways	Total	Expected	Hits	Raw P	Holm P	FDR
Glucose-Alanine Cycle	13	0.156	2	0.00954	0.935	0.522
Alpha Linolenic Acid and Linoleic Acid Metabolism	17	0.204	2	0.0162	1	0.522
Alanine Metabolism	17	0.204	2	0.0162	1	0.522
Transfer of Acetyl Groups into Mitochondria	22	0.263	2	0.0266	1	0.522
Warburg Effect	57	0.683	3	0.0266	1	0.522
Phenylalanine and Tyrosine Metabolism	27	0.323	2	0.0391	1	0.585
Urea Cycle	28	0.335	2	0.0418	1	0.585
Gluconeogenesis	33	0.395	2	0.0565	1	0.692
Propanoate Metabolism	42	0.503	2	0.0868	1	0.85
Lactose Degradation	9	0.108	1	0.103	1	0.85
Glutamate Metabolism	48	0.575	2	0.109	1	0.85
Malate-Aspartate Shuttle	10	0.12	1	0.114	1	0.85
Trehalose Degradation	11	0.132	1	0.125	1	0.85
Taurine and Hypotaurine Metabolism	12	0.144	1	0.135	1	0.85
Phosphatidylethanolamine Biosynthesis	12	0.144	1	0.135	1	0.85
Glycine and Serine Metabolism	59	0.707	2	0.154	1	0.85
Valine, Leucine and Isoleucine Degradation	59	0.707	2	0.154	1	0.85
Phosphatidylcholine Biosynthesis	14	0.168	1	0.156	1	0.85
Arachidonic Acid Metabolism	67	0.802	2	0.189	1	0.855
Spermidine and Spermine Biosynthesis	18	0.216	1	0.196	1	0.855
Butyrate Metabolism	19	0.228	1	0.206	1	0.855
Lactose Synthesis	19	0.228	1	0.206	1	0.855
Glutathione Metabolism	20	0.24	1	0.216	1	0.855
Threonine and 2-Oxobutanoate Degradation	20	0.24	1	0.216	1	0.855
Betaine Metabolism	21	0.251	1	0.226	1	0.855
Glycolysis	23	0.275	1	0.244	1	0.855
Glycerolipid Metabolism	25	0.299	1	0.263	1	0.855
Cysteine Metabolism	26	0.311	1	0.272	1	0.855
Plasmalogen Synthesis	26	0.311	1	0.272	1	0.855
Mitochondrial Beta-Oxidation of Long Chain Saturated Fatty Acids	28	0.335	1	0.29	1	0.855
Phospholipid Biosynthesis	29	0.347	1	0.298	1	0.855
Folate Metabolism	29	0.347	1	0.298	1	0.855
Lysine Degradation	30	0.359	1	0.307	1	0.855
Ammonia Recycling	31	0.371	1	0.316	1	0.855
Citric Acid Cycle	32	0.383	1	0.324	1	0.855
Amino Sugar Metabolism	33	0.395	1	0.332	1	0.855
Beta-Alanine Metabolism	34	0.407	1	0.341	1	0.855
Nicotinate and Nicotinamide Metabolism	35	0.419	1	0.349	1	0.855
Aspartate Metabolism	35	0.419	1	0.349	1	0.855
Fatty Acid Biosynthesis	35	0.419	1	0.349	1	0.855
Galactose Metabolism	38	0.455	1	0.373	1	0.891
Sphingolipid Metabolism	40	0.479	1	0.388	1	0.899
Methionine Metabolism	42	0.503	1	0.404	1	0.899
Histidine Metabolism	42	0.503	1	0.404	1	0.899
Pyruvate Metabolism	47	0.563	1	0.44	1	0.958
Arginine and Proline Metabolism	52	0.623	1	0.474	1	1
Pyrimidine Metabolism	57	0.683	1	0.507	1	1
Tryptophan Metabolism	59	0.707	1	0.519	1	1
Bile Acid Biosynthesis	65	0.778	1	0.555	1	1
Tyrosine Metabolism	70	0.838	1	0.583	1	1
Purine Metabolism	73	0.874	1	0.599	1	1
